# Breast Cancer Leptomeningeal Metastases on Spinal Epidural Lipomatosis

**DOI:** 10.3390/diagnostics14141496

**Published:** 2024-07-12

**Authors:** Alice Rossi, Giada Sancini, Elena Amadori, Patrizia Cenni, Michela Palleschi, Andrea Prochowski Iamurri

**Affiliations:** 1 Radiology Unit, IRCCS Istituto Romagnolo per lo Studio dei Tumori (IRST) “Dino Amadori”, 47014 Meldola, Italy; 2 Department of Medical Oncology, IRCCS Istituto Romagnolo per lo Studio dei Tumori (IRST) “Dino Amadori”, 47014 Meldola, Italy

**Keywords:** MRI, breast cancer, spinal epidural lipomatosis

## Abstract

We present a case of breast cancer metastases superimposed on epidural lipomatosis and although none of these findings are considered rare, their coexistence leads to unique image findings, and as far as we know there are no other cases like this in literature.

A 58-year-old woman with metastatic breast cancer (MBC), with bone metastases since 2013, presented to our department with progressive neuropathic symptoms in the lower part of her torso and legs, along with intermittent sciatic and crural pain. Following the diagnosis of MBC, the patient underwent multiple lines of endocrine therapy, including CDK4/6 inhibitors, such as palbociclib, and chemotherapeutic agents, such as paclitaxel and capecitabine/vinorelbine. Despite these interventions, she continued to experience disease progression. In particular, over the past 3 months, the patient reported the development of paresthesia and dysesthesia in the sub-mammary regions with radiation to the legs. The patient also experienced easy fatigability during walking, with worsening symptoms, until an MRI was ordered to rule out spinal cord compression or other pathologies. Therefore, a contrast-enhanced magnetic resonance scan of the spine was obtained, which demonstrated a thick posterior epidural lipomatosis with compression of the dural sac (Figure 1). Additionally, numerous secondary lesions were observed within the dural sac, adding further compression, with a IV grade of stenosis at the D3 and D7–D8 levels (Figure 1 and Figure 2), where the spinal cord appeared compressed, with the high signal on T2-weighted sequences suggestive of spinal cord distress. The high-grade cord compressions highlighted at the MRI explained the neuropathic symptoms in the lower part of the torso, and the patient was then referred for palliative radiotherapy treatment to the involved thoracic spinal segment. Breast cancer in Europe is the leading cause of cancer-related death in women. Progression to MBC occurs in about 20–30% of patients without metastases. MBC is still an incurable disease and when diagnosed; the 5-year survival rate is around 38%, but survival improvements have been reported with appropriate therapeutic strategies [1]. Carcinoma-related leptomeningeal metastasis is a relatively rare event (5% of breast cancer patients); however, MBC remains the most common etiology of leptomeningeal carcinomatosis. The prognosis remains poor, with a median survival of 4 months [2]. Epidural lipomatosis is a rare clinical condition represented by an abnormal deposit of fat in the spinal canal. It may press on the spinal cord and nerves, causing neurological deficits, and it can be classified into four main categories based on pathogenesis: exogenous steroid use (55.3%), obesity (24.5%), idiopathic (17%), and endogenous steroid hormone disorder (3.2%) [3]. 

## Figures and Tables

**Figure 1:**
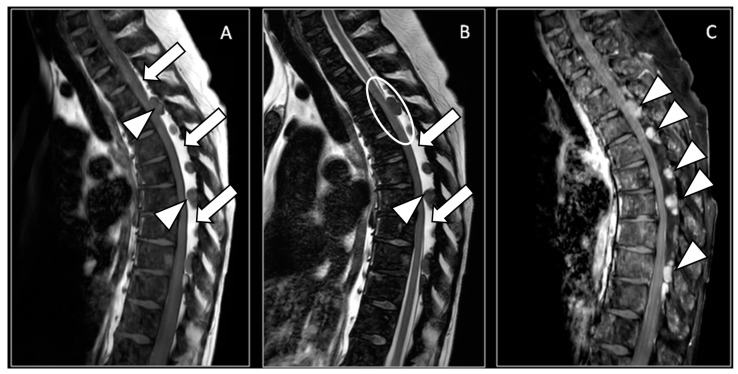
(**A**) Sagittal magnetic resonance T1-weighted image; (**B**) sagittal magnetic resonance T2-weighted image; (**C**) sagittal magnetic resonance T1 fat-suppressed weighted image after endovenous contrast media administration: a thick posterior epidural lipomatosis (white arrows), with high signal in A and B and low signal in C, that compresses the dural sac, in particular in the dorsal tract. The epidural lipomatosis should not be confused with the spinal cerebrospinal fluid, which demonstrates a high signal only in B and can be appreciated only in the cervical and the distal dorsal tracts. Within the lipomatosis, there are multiple solid nodulations, with a low signal in (**A**,**B**), characterized by a high signal in (**C**) (white arrowheads), due to pathological contrast enhancement. One nodulation adds further cord compression at the D3 level, where the spinal cord appears compressed with a high hyperintensity signal on T2w sequences, suggestive of spinal cord distress (white circle in (**B**)). Note also the diffuse pathological signal in all sequences of the vertebral bone marrow.

**Figure 2:**
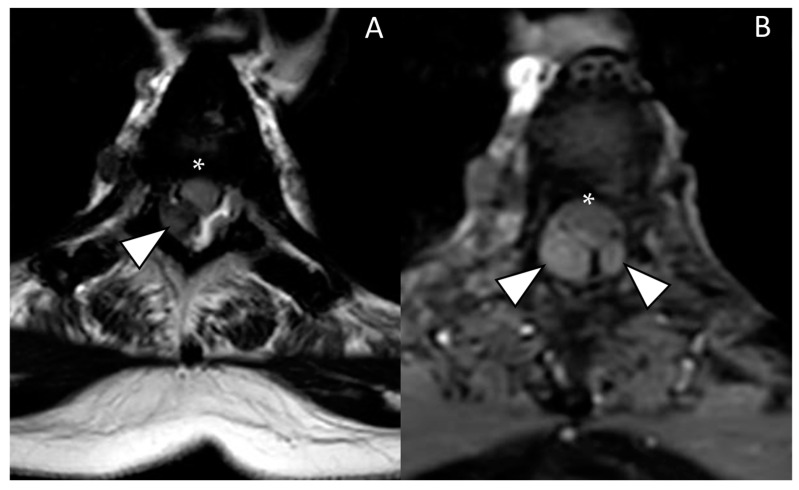
(**A**) Axial magnetic resonance T1-weighted image; (**B**) axial magnetic resonance T1 fat-suppressed weighted image after endovenous contrast media administration: axial images show the malignant solid nodules (white arrowheads) on the epidural lipomatosis that compress the spinal cord (white asterisk). Note also the diffuse pathological signal of the vertebral bone marrow.

## Data Availability

No new data were created or analyzed in this study. Data sharing is not applicable to this article.
